# Seasonal Change in Distribution and Heat Coma Temperature of Oceanic Skaters, *Halobates* (Insecta, Heteroptera: Gerridae)

**DOI:** 10.3390/insects9040133

**Published:** 2018-10-05

**Authors:** Tetsuo Harada, Mitsuru Nakajo, Takahiro Furuki, Noritomo Umamoto, Masatoshi Moku, Takero Sekimoto, Chihiro Katagiri

**Affiliations:** 1 Laboratory of Environmental Physiology, Graduate School of Integrated Arts and Sciences, Kochi University, Kochi 780-8520, Japan; tfuruki9269@gmail.com (T.F.); uma919acvd@gmail.com (N.U.); jm-t.sekimoto@kochi-u.ac.jp (T.S.); 2 Laboratory of Science Education, Graduate School of Integrated Arts and Sciences, Kochi University, Kochi 780 8520, Japan; Mit-na@kochi-u.ac.jp; 3 Atmosphere and Ocean Research Institute, The University of Tokyo, 277-8524 Kashiwa, Japan; 4 Faculty of General Education, Tokyo Denki University, Tokyo 120-0034, Japan; lp@lowtem.hokudai.ac.jp

**Keywords:** population density, habitation temperature range, heat coma temperature, oceanic skaters, season

## Abstract

A series of studies were conducted during two cruises between Tokyo and Honolulu in September 2010 and from February to March 2012. The aims of the studies were to (1) compare the distribution of three species of *Halobates* oceanic skaters, *H. germanus, H. micans*, and *H. sericeus,* with respect to their temperature limits; (2) identify the lower temperature limit of *H. sericeus*, the species that displays the widest distribution range (40°N–35°S) latitude; and (3) test the hypothesis that *H. sericeus* can change their temperature tolerance to adapt to seasonal changes in sea surface temperatures. The heat coma temperature (HCT) was measured during the two cruises and the values were compared between the two populations of *H. sericeus*. The species collected in September 2010 were *H. germanus*, *H. micans*, and *H. sericeus*. *H. sericeus* was dominant, occupying more than 90% of the collecting sites. *H. germanus* and *H. micans* were collected in the northern and western part of the cruise track (29–34°N, 141–151°E), and not in the southern and eastern part. The population density of these two species was 9000–150,000/km^2^ in the first cruise, which took place in summer. On the other hand, *H. sericeus* was collected throughout the cruise track during that cruise. The population density of *H. sericeus* was relatively high, at 4000–310,000/km^2^, in the southern and eastern part of the cruise track (19–29°N, 152°E–165°W). In February and March 2012, only *H. sericeus* was collected at a density of 17,000–80,000/km^2^ and only in the eastern and southern part, at 25°–28°N, 169°E–178°W. No *Halobates* oceanic skaters were found in the western or northern part (30°N and further north, 159°E and further west) during that cruise. The lower limit for the inhabitation of sea surface temperatures appeared to be 27.8 °C or slightly lower for *H. germanus* and *H. micans*, but was 22.1 °C or slightly lower for *H. sericeus*. *H. sericeus* specimens, mostly adults, that had been collected during the two cruises were used in heat coma experiments. Summer specimens showed significantly higher heat coma temperatures (HCTs) than the winter specimens. This difference in HCTs may be the result of relatively long term temperature acclimation in the summer or winter for the adults that inhabit the temperate and subtropical areas along the cruise tracks between Tokyo and Honolulu in the Pacific Ocean. This temperature plasticity of *H. sericeus* may be related to the wider latitude area inhabited by this species (main range: 40°N–25°S).

## 1. Introduction

Insects are found everywhere from the Antarctic to the tropical terrestrial zone. This wide distribution means that they are highly adapted to a wide ambient temperature range [1]. Water striders in the family Gerridae can tolerate a lower ambient temperature limit of –3 °C [2]. The lower ambient (water surface) temperature limit of oceanic skaters is said to be around 25 °C for their distribution [3,4,5]. However, no detailed sampling has been carried out on their distribution related to sea surface habitat temperature in *Halobates*. One species in particular, *H. sericeus*, shows a latitude distribution of 40°N–35°S, which is wider than that of other oceanic skaters [4]. *H. sericeus* may be able to inhabit the subtropical oceanic area (around 25°N) even in winter, with surface sea temperatures of around 20 °C–25 °C, which may be the critical range for the distribution of this species. However, no data have been found on the critical inhabiting area showing the lower limit temperature of the oceanic skaters in the winter season. Therefore, the first aim of this study was to estimate the critical lower limit temperature for distributions of *H. sericeus* in winter. To determine this, the distribution of *H. sericeus* and surface water temperatures in winter were examined around the cruise tracks between Tokyo and Honolulu in the subtropical and warm-temperature zones of the Pacific Ocean.

*Halobates* comprises 68 species that are mostly coastal species; only six species inhabit the open ocean [4,6]. Three of these species of oceanic skaters (Heteroptera, Gerridae), *H. germanus, H. micans,* and *H. sericeus* inhabit the Pacific Ocean. These species differ in the latitude range of their distribution, which is 0°–20°S, 15°N–15°S, 40°N–35°S, respectively, in the Pacific Ocean [7]. Based on this difference in the latitude distribution, *H. sericeus* has been hypothesized to have a lower limit of ambient temperature for inhabiting and a resistance to a wider temperature range than the other two species. However, this hypothesis has yet to be investigated. The distributions of the three oceanic skater species, *H. germanus*, *H. micans*, *H. sericeus*, in the East China Sea have already been reported on by Harada [5]. In Harada [5], *H. sericeus* specimens were collected at stations with a wide range of surface water temperatures (22.1–27.0 °C). Conversely, few or no *H. micans* or *H. germanus* were collected at stations with surface water temperatures between 22.1 °C and 22.5 °C. This suggests that these two species may have difficulty inhabiting areas where the water temperature is lower than 23 °C. However, no comparative studies have been conducted on the critical limits for the distribution of these three species of oceanic skaters throughout a wide longitudinal area of the Pacific Ocean, both in summer and winter in subtropical and warm temperate zones. The second aim of this study was therefore to examine these critical limits in these three species.

In ectotherms, the ambient temperature in the habitats of insects is related to temperature tolerance [8,9,10]. Such physiological flexibility appears to be one of the main factors determining their survival. In particular, the ability to acclimate to a new temperature state may be one of the key processes by which organisms can adapt to increased temperatures caused by global warming [8,9,10]. Many studies have been conducted to measure the acclimation capability of marine ectotherms to higher temperatures, testing the beneficial acclimation hypothesis [10,11,12]. Improvement in performance at a higher temperature may be an effective indicator of whether or not a species can achieve a higher thermal tolerance and adapt to a new habitat [12,13,14,15]. In the case of oceanic skaters inhabiting temperate zones, seasonal change in sea surface temperature is dynamic. To understand how oceanic skaters can invade a new specific environment, it is important for us to understand how organisms adapt to a new temperature condition in their environment. The seasonal change of sea surface temperature occurs naturally in the temperate habitat of oceanic skaters. Adults of *H. sericeus* are hypothesized to acclimatize to the seasonal change in their habitat temperature. This study was performed to test this hypothesis, as the third aim.

## 2. Materials and Methods

### 2.1. Sampling

**Figure 1:**
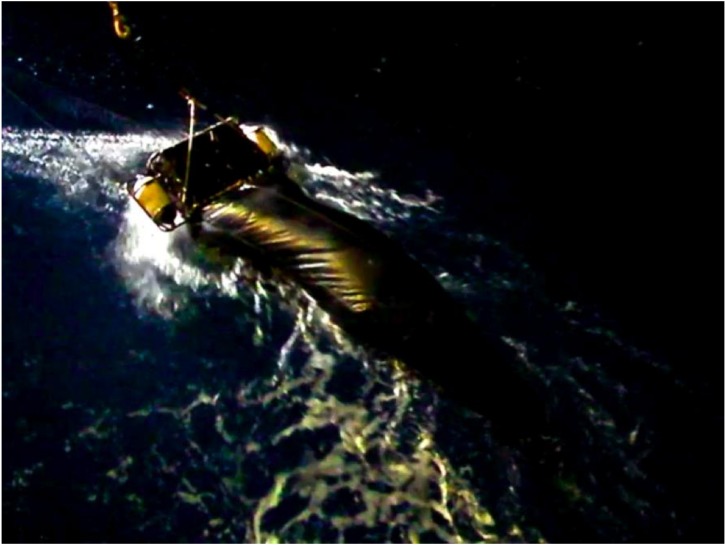
The nets used for sampling were Neuston nets modified from Manta type nets with a width of 1.3 m and length of 6 m with buoys on both sides.

Sampling was performed from 1 September to 13 September 2010 (cruise no. KH-10-04) and from 23 February to 4 March 2012 (KH-12-02) with a Neuston net (6 m long and a diameter of 1.3 m; modified from the ‘Manta style’; Figure 1). The Neuston net was towed for 15 min. Between six and eight tows were performed at each site in the summer and three tows in the winter.

**Figure 2:**
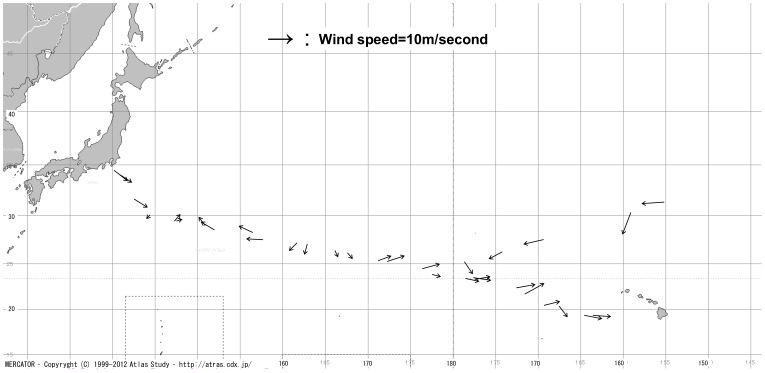
Schematic representation of the cruise routes (KH-10-04 and KH-12-02) by the RV Hakuho Maru. Arrows show the wind direction and wind speed at each sampling point.

Sampling was performed at 26 stations in summer and six stations in winter along the cruise track between Tokyo and Honolulu in the subtropical and temperate Pacific Ocean (Figure 2). Samples were taken from the starboard side of the ship, RV Hakuho Maru (3991 t), owned by the Japan Agency for Marine-Earth Science and Technology (JAMSTEC). The towing was performed exclusively at night, at a ship speed of 2.0 knots. The surface area that was swept by the Neuston net was evaluated as flow-meter value × 1.3 m (width of the Neuston net). During the sampling, a small bucket (15 cm diameter × 20 cm height) attached to a rope was thrown down onto the sea surface from the starboard side and the surface sea water was collected. The bucket was then pulled up onto the deck and a mercury thermometer was used to measure the temperature of the water and the air temperature on deck (Table 1).

### 2.2. Treatment of Specimens for Heat Coma Experiment

As *H. sericeus* was collected in the subtropical zone (mainly in 24°N–27°N) and was the dominant species in this area, most specimens used to examine heat coma temperature in this study were from this species. Specimens trapped in the cod end of the Neuston net were transferred to a round transparent aquarium on the deck of the ship. They were then quickly moved to a dry laboratory near the deck on the ship. The room temperature in the dry laboratory was kept within 2 °C of the surface water temperature in the sea area where the sampling was performed. The collected specimens were mostly paralyzed from the force of the trailing and were quickly cleaned using paper towel to remove water. Any jellyfish remains on the specimens were removed by hand in the dry laboratory. Sixty to seventy percent of specimens recovered from paralysis within 20 min post collection.

For the first eight to twelve hours after collection, the specimens were kept in white cubic aquaria (30 × 30 × 40 cm) to allow acclimation to the conditions inside the laboratory. The aquaria were filled with natural sea water from the ocean that was filtered by mesh with a pore size of 90 micrometers to remove most organic substances and cells. The aquaria were designed to shut out external disturbances. Each aquarium contained between ten and thirty adults. Both the room temperature and sea water temperature in the aquaria were kept at values close to those of the ocean surface temperature (26 ± 2 °C for winter and 29 ± 2 °C for summer). 

Food was restricted to avoid contents in the alimentary canal of the oceanic skaters, which have been reported to affect the super cooling points of insects [16]. Oceanic skaters can survive for 90 to 100 h on average without feeding, and in this experiment, the oceanic skaters were deprived of food for at least 12 h before and during the heat coma temperature experiment.

Just before the heat coma temperature experiments, specimens were transferred from the cubic aquaria to transparent circular experimental aquaria filled with 2 L of filtered sea water which were covered by corrugated cardboard as a heat insulating material [6,17,18,19]. 

### 2.3. Heat Coma Experiment

Heat coma temperatures (HCTs) of the summer specimens were measured and compared to those of the winter specimens. Ten adult specimens were transferred to the experimental aquaria where the water temperature was increased by 1 °C per hour. The water temperature was controlled very precisely with an electric heater (Auto-heater 55, GEX Co. Ltd., Osaka, Japan) that could be switched on and off manually to keep values within ± 0.3 °C. The water was also stirred by hand to keep the water temperature consistent using a 10 cm long, 5 mm wide hollow tube with a 3 cm diameter stone ball at the end. The experiments were performed by researchers who had been trained for at least 36 h.

HCTs are defined as the temperature at which the ventral surface of the specimen’s body sticks to the sea water film and the ability to skate is lost, or abnormal postures on the sea water are observed (such as one leg sinking into the water, being upside-down, or a mid-leg thrust behind and stuck to a hind leg). Semi-heat coma temperatures (SHCTs) are defined as the temperature at which the oceanic striders show little or no movement on the water surface for more than 3 s. SHCTs occur just prior to heat coma. The HCTs and SHCTS were recorded for each specimen and used as heat tolerance evaluation values. In most cases, semi-heat coma occurred first and was followed by heat coma. Oceanic skaters were quickly removed from the experimental arena just after the measurement of HCTs. 

### 2.4. Statistical Analysis

The data in this study were statistically analyzed by Mann-Whitney *U*-tests for the analysis on the number of specimens collected in two temperature categories: (1) greater than 28 °C; (2) less than 28 °C. This cutoff was chosen because, in the tropical Pacific Ocean habitat for oceanic skaters, the surface temperature of 28 °C is hypothesized to be a typical lower limit of water surface temperature. For example, 28 °C could occur when it is rainy in the summer or in subtropical regions (Harada et al., unpublished). 

Seasonal differences between summer and winter in HCTs and SHCTs were tested using the Mann-Whitney *U*-test. Another analysis of co-variance (ANCOVA) was also used for the analysis of the effects of sex and season on HCTs and HSCTs. All analyses were performed with SPSS 12.0 statistical software.

## 3. Results

### 3.1. Distribution

The results of the samplings are described in Table 1. These samplings indicated that four species, *H. germanus*, *H. micans*, *H. sericeus*, and *H.* sp. inhabited the region during the summer (*H.* sp.: possibly a new species inhabiting the open ocean; body length: 3.5–4.6 mm, longer thorax relative to body width; a formal description is in preparation). The relationship between the number of specimens collected and surface temperature at the sampling point was not examined for *H.* sp., as the number of sites from which this species was collected was limited. The only location where all four species were collected was at 30°–34°N and 140°–144°E along the western part of the cruise track during the summer cruise, where *H. sericeus* was dominant. 

In winter, only *H. sericeus* was collected in the area from 19°–29°N and 147°E–163°W along the cruise track. Many larvae of this species were collected at several subtropical stations (Table 1). In the southern area, from 24°30′N, 177°32′W to 26°27′N, 174°15′E, between 68 and 198 specimens were collected over three stations. At a station located at 27°42′N, 169°24′E, only 42 *H. sericeus* specimens were collected. At two stations located further north (30°15′N, 159°04′E and 31°27′N, 154°07′E), no *H. sericeus* specimens were collected. No *H. micans* and *H. germanus* specimens were collected in winter.

More *Halobates* specimens were collected in summer than in winter (summer: 53.2 ± 52.0 [all 177 sweepings]; winter season: 25.5 ± 18.5 [12 sweepings] (Mann-Whitney U-test: n = 189, z = −1.674, p = 0.084). In the winter, samplings were also performed at two additional points further northwest (30–31°N, 154–159°E), but no specimens were collected (Table 1). A slightly higher ratio of female specimens were collected in both seasons (summer: female-ratio for adults, 0.624 ± 0.163, n = 177 sweepings; winter: 0.613 ± 0.152, n = 12, U-test: n = 189, z = −0.843, p = 0.399). In winter, females showed a slightly higher resistance to high temperatures than males (Table 2). However, slightly higher female survival ratio in *H. sericeus* may not have affected this minimal gender difference in temperature resistance.

*H. sericeus* was the dominant species at more than 90% of all sampling sites in summer (Table 1). *H. germanus and H. micans* were collected only in the northern and western part (29–34°N, 141–151°E) in the summer cruise, with a population density of 9000–150,000/km^2^. On the other hand, *H. sericeus* was collected along the entire track. The population density of *H. sericeus* was 4000–310,000/km^2^ in the southern and eastern part of this track (19–29°N, 152°E–165°W). In the winter cruise, only *H. sericeus* was collected, and this species appeared only in the eastern part (25°–28°N, 169°E–178°W) of the cruise track with the density of 17,000-80,000/km^2^. However, the estimate of the number of insects per km^2^ could be an overestimation as *Halobates* is known to have an uneven distribution. There were no oceanic skaters in the western and northern part (30°N or northern, 159°E or western) of this winter cruise track. The lower temperature limit for inhabitation was 27.8 °C for *H. germanus* and slightly lower for *H. micans.* It was even lower for *H. sericeus*, at 22.1 °C.

**Figure 3:**
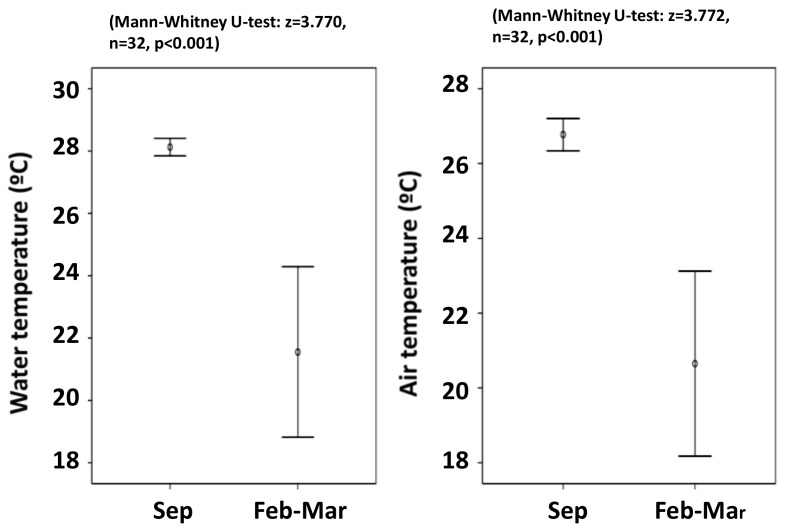
Difference in sea surface temperature along the cruise track between Tokyo and Honolulu between September 2010 and February–March 2012.

In winter, the *H. sericeus* habitat was limited to between 27°41–2′N, 169°23–4E and 30°15′N, 159°04–5′E, which has surface water temperatures of 22.1 °C and 18.9 °C, respectively. Seasonal variation in the ocean water surface temperature and the air temperature is shown in Figure 3. There was a seasonal difference of about 6 °C between the summer and winter cruise (Figure 3). 

### 3.2. Relationship between Water Temperature and the Number of Individuals Collected

**Figure 4:**
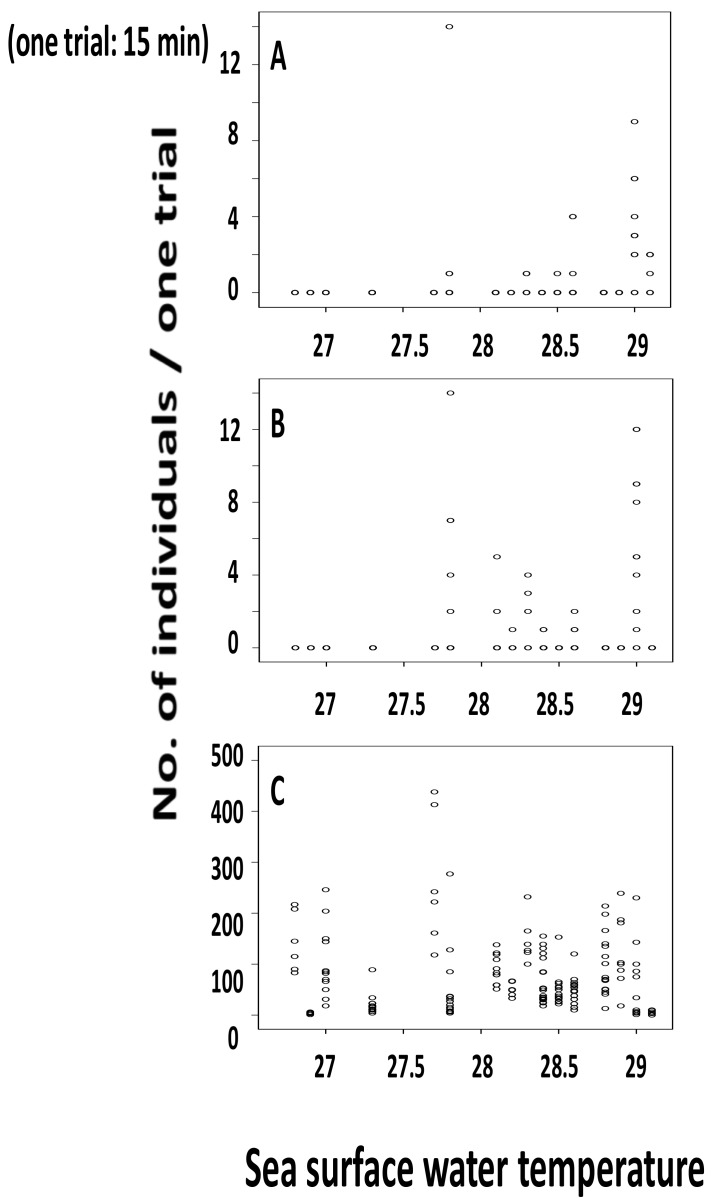
The relationship between sea surface water temperature at the collection sites and the individual number of *Halobates micans* (**A**), *H. germanus* (**B**), and *H. sericeus* (**C**) collected on the route Tokyo–Honolulu during the cruise KH-10-04-Leg 1 in September 2010.

The surface water temperature ranged from 26.6 °C to 29.1 °C in the summer cruise and 17.7 °C to 23.7 °C in the winter cruise (Table 1). Individuals of *H. micans* (Figure 4A) and *H. germanus* (Figure 4B) were collected only in the summer cruise and only from areas where the surface temperature was more than 27.8 °C. None of either species was collected from any sampling area in winter. 

**Figure 5:**
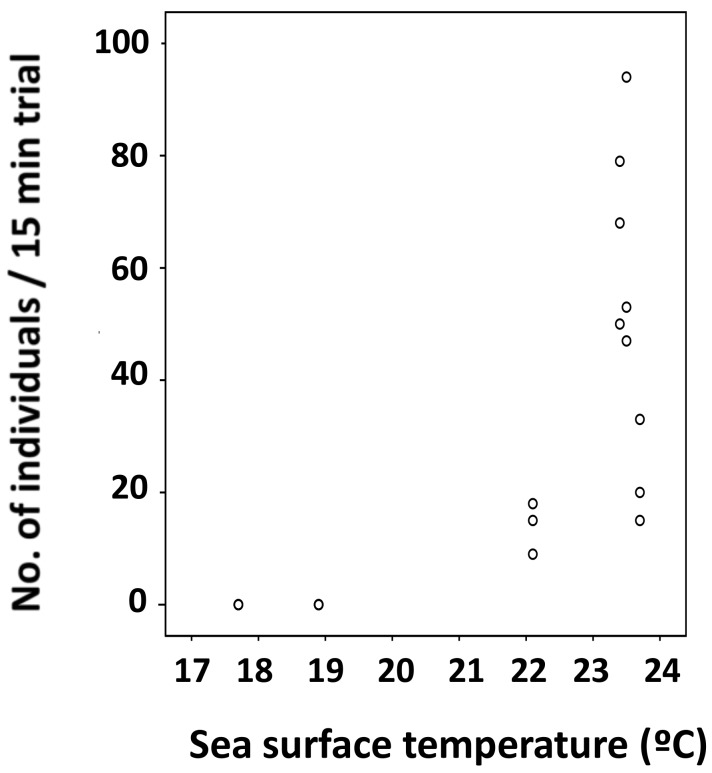
Relationship between sea surface temperature at the collection sites and the individual number of *Halobates sericeus* specimens collected on the route (Tokyo–Honolulu) during the cruise KH-12-01-Leg 2 in February and March 2012.

Many *H. sericeus* specimens were collected throughout the whole temperature range in summer (Figure 4C). In winter, specimens of this species were collected only from areas where the surface water temperature was more than 22 °C (Figure 5). 

**Figure 6:**
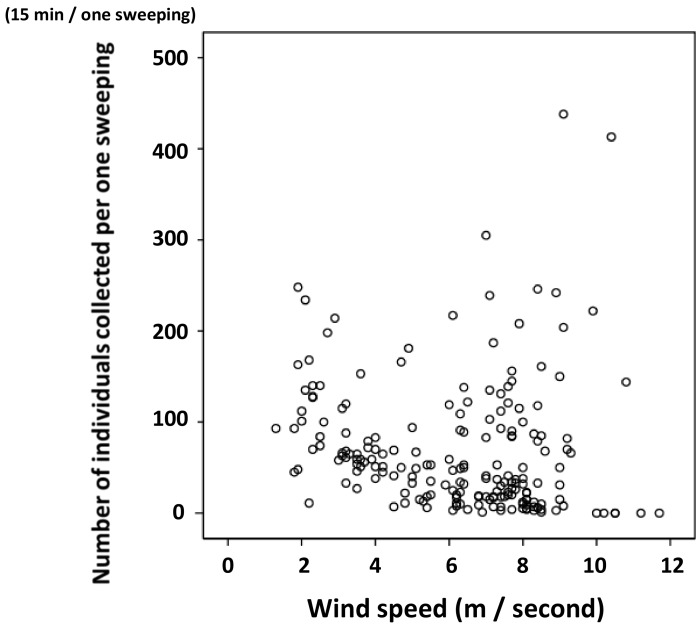
The relationship of the number of individual oceanic skaters to wind speed during collection. (Pearson’s correlation analysis: *n* = 195, *r* = −0.116, *p* = 0.108).

The relationship between the number of individuals collected in one sample for 15 min and wind speed is shown in Figure 6. Although there was no significant correlation in these cruises, two large samples of more than 400 individuals were collected when the wind speed was more than 9 m/second. When many individuals are floating nearby the sampling net, strong winds could sweep them into the net as they are so light. 

### 3.3. Statistical Analysis on Number of Individuals Collected and Surface Water Temperature

Significantly higher numbers of individuals were collected per 15 min trail by the Neuston net from those stations with water surface temperatures of more than 28.0 °C (*H. micans*, mean ± SD (number of trials): 0.54 ± 1.75 (135)); *H. germanus*: 0.92 ± 2.52 (135); *H. sericeus*: 77.8 ± 73.9 (135)) than those from those stations with temperatures less than 28.0 °C (*H. micans*: 0.0 ± 0.0 (42); *H. germanus*: 0.0 ± 0.0 (42); *H. sericeus*: 57.9 ± 68.2 (42)). The results of the Mann-Whitney *U*-tests on the number of collected individuals between the two categories ((1) temperature range more than 28 °C; and (2) less than 28 °C) were as follows: *H. micans*: *z* = −2.78, *p* = 0.005; *H. germanus*: *z* = −3.058, *p* = 0.002; *H. sericeus*: *z* = −2.457, *p* = 0.014.

### 3.4. Semi-Heat Coma Temperatures (SHCTs) and Heat Coma Temperatures (HCTs)

Semi-heat coma temperatures (SHCTs) and heat coma temperatures (HCTs) ranged from 26 °C–38 °C and from 29 °C–39 °C, respectively (Ta. In specimens obtained in summer, the averages (±SD [n]) of the HSCTs and HCTs were 36.0 °C (±2.1 (128)) and 36.6 °C (±1.3 (128)), respectively (Table 2). These values were significantly higher in summer than those from specimens collected in the winter (HSCTs: 33.7 ± 2.0 (35) °C; HCTs: 35.1 ± 0.9 (35) °C) (ANCOVA (HCTs or SHCTs as dependent variance; season: independent variance; sex: covariance), HCTs, on the season, *df* = 1, *F* = 41.1, *p* < 0.001; SHCTs, *df* = 1, *F* = 33.6, *p* < 0.001; Table 2). There were no significant differences in the SHCTs between sexes in either the summer or winter and no differences in HCTs between the sexes in the summer (Mann-Whitney U-test: summer SHCTs, *z* = −0.878, *p* = 0.380, Winter SHCTs, *z* = −0.978, *p* = 0.328, summer HCTs, *z* = −1.556, *p* = 0.120). Females showed significantly higher HCTs than males in winter (*z* = −3.035, *p* = 0.002; Table 2).

## 4. Discussion

### 4.1. Distribution and Habitat Temperature

In the present study, *H. sericeus* had a low temperature limit of 22 °C for its winter distribution in the subtropical Pacific Ocean, showing a high resistance to lower temperatures. In contrast, the other three species, *H. germanus*, *H. micans*, and *H.* sp., were not present in this part of the ocean even in an area with a surface temperature as high as 27 °C in September. In the case of *H. germanus* and *H. micans*, the lower temperature limit for inhabitation may be between 27.5 °C and 28 °C based on the results of collection in the summer.

Cheng [3] stated that the occurrence and abundance of oceanic skaters can be primarily controlled by surface sea water temperatures. The optimal temperature for oceanic skaters was estimated to be 25–30 °C [3,20]. Moreover, Andersen and Cheng [4] claimed that the lower limit of the surface water temperature for survival of oceanic skaters was around 25 °C. The present study showed that the critical lower temperature for inhabitation by *H. sericeus* may be as low as 21 °C. Meanwhile, the critical lower temperature for inhabitation by the other two species, *H. germanus* and *H. micans*, may be higher, at around 28 °C, which is even higher than the value discussed by Andersen and Cheng [4].

Furuki et al. [21] recently showed that *H. sericeus* was a cool hardy species with a cool coma temperature in the range of 12–24 °C. The lower temperature limit for the cool coma of *H. sericeus* [21] may also be related to the lower temperature of its distribution which ranges widely in latitude from 5°N to 42°N and also to 27°S [22,23]. The Northern Equatorial Current and the *Kuroshio* Current may transfer *H. sericeus* from lower to higher latitudes in the northern hemisphere [24]. There is a wider temperature range of the surface sea water from around 30 °C to 22 °C in subtropical and temperate zones. This wide range of surface temperatures in their habitats may be related to *H. sericeus’* strong resistance to lower temperatures of 12–24 °C [21]. The two other species, *H. micans* and *H. germanus*, could also be transferred to a higher latitude area by the *Kuroshio*. However, the species’ relatively lower resistance to temperatures under 25 °C may be related to their absence at higher latitudes of 30°–40°N [4,5].

### 4.2. Seasonal Change of Heat Tolerance as a Possible Temperature Acclimation Phenomenon

One hypothesis in this study was that seasonal changes in sea surface temperatures in habitats could alter the temperature tolerance range in oceanic skaters due to acclimation to habitat temperatures. The results of the present study support this hypothesis. Namely, in the warm temperate zone and subtropical zone of the Pacific Ocean between Tokyo and Honolulu, the *H. sericeus* specimens collected in summer showed a stronger resistance to higher temperature than the specimens collected in the same habitat in winter. This could mean that temperature acclimation effects for this species occur in summer and winter seas. In actuality, the water surface temperature ranged from 27.8 °C to 29.1 °C when the experimental specimens were collected in summer and 22.1 °C to 23.4 °C in the winter. 

Many studies have been conducted on cold hardiness in insects [1,25]. Similarly, several studies have been conducted on heat-hardiness in ectotherms. In one example, four polar species of gastropods demonstrated relatively clear temperature acclimation in the Arctic [26]. In a study by Richard et al. [26], acclimation to 7.1 °C over two months produced reductions in acute upper temperature limits (critical thermal maximum temperature with temperature increasing by 1 °C per day), while acclimation to 10.3 °C produced increases in acute upper temperature limits. Findings on marine gastropods may be similar to the results of this study on temperature acclimation for the temperature limit of marine invertebrates. This acclimation in the oceanic skaters, *H. sericeus* may be an ability developed for the range for temperature tolerance to adapt seasonal changes.

In the present study, we examined heat coma temperature, which is an index of heat tolerance used on water surface species [6,17,18]. One of other indices of heat tolerance that have been used on water surface species is lethal temperature (the temperature at which death occurs due to heat or cold). Moreover, other indices have been used to measure heat tolerance in terrestrial insects. For example, heat acclimation promotes “dropping behavior” to avoid heat stress in aphids [27]. Various studies have used the critical thermal maximum (CTmax), knockdown temperature/time, or heat coma temperature as ways to evaluate thermal tolerance in insects. These indices are defined as the temperatures at which the specimens begin to lose muscle control, stop walking, or stop moving their legs or antennae [28,29,30,31] or the temperature at which the ventral surface of the body begins to be submersed in the water surface in the case of oceanic skaters [6,17,18,19]. Most of these studies have shown that exposure to high temperature makes physiological heat tolerance harder as an acclimation. In this study, a type of water-temperature acclimation could also be produced during exposure to high or low temperatures in their habitat during the growth phase and while living as an adult as well as in the oceanic skater, *H. sericeus*. Adaptation to seasonal temperature variation of about 6 °C suggests that this species may be capable of adapting to higher ambient temperatures. This adaptive power would be advantageous because temperatures might increase in the future due to global warming.

This study had some limitations. One is that our measure of field temperature gradient may be conflated with distance from landfall (island), which may limit oviposition to floating debris. 

Another is that the absence of two species (*H. germanus* and *H. micans*) in February could be due to diapause of the egg or adult stage in the area studied [7]. This is a life history pattern that may have evolved to survive low prey availability, low temperature, or both. Some gerrids such as *Metrocoris histrio* (Harada et al. unpublished) diapause as eggs, and some diapause as adults on land, yet the details about diapause in *Halobates* remain unknown.

## 5. Conclusions

The lower limit for the inhabitation of sea surface temperatures appeared to be 27.8 °C or slightly lower for *H. germanus* and *H. micans* in a summer cruise, but was 22.1 °C or slightly lower for *H. sericeus* in winter cruise between Tokyo and Honolulu. Summer adult specimens in *H. sericeus* showed significantly higher heat coma temperatures (HCTs) than the winter specimens and it might be the result of one kind of temperature acclimation.

## Figures and Tables

**Table 1:** The oceanic skaters, *Halobates* were collected at sites along the cruise tracks between Tokyo and Honolulu in September, 2010 (A-Cruise: KH-10-04-Leg 1), and in February and March 2012 (B-Cruise: KH-12-01-Leg 2). T: Total number of individuals collected; L: Larvae, A: Adults; *H.g.*: *Halobates germanus*; *H.m.*: *Halobates micans*; *H.s.*: *Halobates sericeus*; *H*. spp.: *Halobates* spp.; EG: Number of eggs; E: Number of exuviae; WT: Water temperature (°C); AT: Air temperature; Time: Time of sampling; Date: Sampling date; SAT: Area of water surface over which the Neuston net was trailed by the ship, R/V HAKUHOMARU (One unit of sampling was performed for 15 min; six (or eight) units in A-Cruise and three units in B-cruise were performed at each sampling site); PD: Population Density (individuals/km²).

Cruise No.	Sampling Sites (Sample No.)	T	L	A	*H.m.*	*H.g.*	*H.s.*	*H*. spp.	EG	E	WT	AT	Time	Date	SAT	PD
KH-10-04	34°43–45′N, 140°14–24′E (1)	645	239	406	16	34	595	0	0	3	27.8	29.8	20:02–22:00	1-Sep	5109	126,248
KH-10-04	34°05–06′N, 141°21–26′E (2)	54	14	40	9	12	32	1	0	5	29.1	26.8	02:09–04:00	2-Sep	6012	8982
KH-10-04	31°11–12′N, 143°41–46′E (3)	91	37	54	7	0	42	42	0	0	29.1	27.3	18:45–20:57	2-Sep	6518	13,961
KH-10-04	30°5–8′N, 144° E30–32′E (4)	754	140	614	29	55	668	2	0	0	29	26.8	02:07–03:55	3-Sep	6506	115,893
KH-10-04	29°38′N, 147°37–40′E (5)	418	147	271	1	0	408	9	0	0	28.5	26.3	18:22–20:26	3-Sep	5316	78,631
KH-10-04	29°36′N, 147°48E (6)	897	109	788	2	9	886	0	0	0	28.3	26.3	01:32–03:15	4-Sep	6081	147,509
KH-10-04	29°03′N, 151°0–4′E (7)	326	65	261	8	4	314	0	0	0	28.6	26.2	17:42–20:04	4-Sep	7167	45,486
KH-10-04	28°49–51′N–152°18–21′E (8)	638	128	510	0	0	638	0	0	0	28.1	24.9	02:07–03:47	5-Sep	5132	124,318
KH-10-04	28°08′N, 156°19–24′E (9)	397	29	368	0	0	397	0	0	0	28.4	27.2	18:40–21:04	5-Sep	5906	67,220
KH-10-04	27°55′N, 157°40–43′E (10)	217	7	210	0	1	216	0	0	0	28.4	26.5	02:05–03:52	6-Sep	4862	44,632
KH-10-04	27°17–18′N, 161°13–17′E (11)	407	39	368	0	0	407	0	0	0	28.8	27.8	18:12–20:44	6-Sep	6360	63,994
KH-10-04	27°0–2′N, 162°45–47′E (12)	287	10	277	0	1	286	0	0	0	28.2	28.4	02:59–04:42	7-Sep	5295	54,202
KH-10-04	26°25–6′N, 166°9–12′E (13)	1145	253	892	0	0	1145	0	0	0	28.8	26.6	18:41–21:04	7-Sep	6878	166,473
KH-10-04	26°10–11′N, 167°39–41′E (14)	383	29	354	1	0	382	0	0	0	28.6	28.7	02:32–04:20	8-Sep	5292	72,373
KH-10-04	25°33–5′N, 171°E–19–24E (15)	988	335	653	0	0	988	0	0	0	28.9	26.3	18:41–21:06	8-Sep	6981	141,527
KH-10-04	25°18–9′N, 172°41–4′E (16)	689	140	549	0	1	688	0	0	0	28.4	27.8	02:01–03:42	9-Sep	5312	129,706
KH-10-04	24°38–42′N, 176°25–9′E (17)	279	81	198	0	0	279	0	0	0	28.5	27.2	18:12–20:34	9-Sep	6967	40,046
KH-10-04	24°23–4′N, 177°E46–9′E (18)	417	173	244	0	7	410	0	0	0	28.1	26.9	01:31–03:11	10A-Sep	4484	92,997
KH-10-04	23°36–8°N, 178°E34–8′E (19)	99	37	62	0	0	99	0	0	0	27.8	24.2	18:43–21:05	10A-Sep	7215	13,721
KH-10-04	23°10–1°N, 177°E14–6′W (20)	1594	685	909	0	0	1594	0	0	0	27.7	27.1	02:29–04:09	10B-Sep	5128	310,842
KH-10-04	22°11–5′N, 173°48–51′E (21)	115	51	64	0	0	115	0	0	0	27.3	26.3	18:42–20:03	10B-Sep	7352	15,642
KH-10-04	21°48–9′N, 173°48–51′E (22)	538	214	324	0	0	538	0	0	0	27	25.9	02:03–03:43	11-Sep	5484	98,104
KH-10-04	20°49–53′N, 169°12–6′W (23)	200	118	82	0	0	200	0	0	0	27.3	26.3	18:11–20:34	11-Sep	7188	27,824
KH-10-04	20°20–23′N, 167°56′W (24)	695	406	289	0	0	695	0	0	0	27	25.7	02:52–04:36	12-Sep	5248	132,431
KH-10-04	19°33–5′N, 164°39–45′W (25)	26	8	18	0	0	26	0	0	0	26.9	26.8	18:38–21:00	12-Sep	6608	3935
KH-10-04	19°39–40′N, 163°48–52′W (26)	858	338	520	0	0	858	0	0	0	26.6	26.4	03:15–05:04	13-Sep	5050	169,901
KH-10-04	In total	13,157	3832	9325	73	124	12,906	54		8					155,451	84,638
Cruise No.	Sampling sites	N	L	A	*H.m.*	*H.g.*	*H.s.*	*H.* spp.	EG	E	WT	AT	Time	Date	SAT	PD
KH-12-1	24°30′N, 177°31–2′W (27)	197	123	74	0	0	197	0	0	2	23.4	22	02:08–02:59	25-Feb	2466	79,886
KH-12-1	25°17′N, 178°57–8′E (28)	68	22	46	0	0	68	0	0	0	23.7	22.4	20:04–20:55	26-Feb	2235	30,425
KH-12-1	26°27–8′N, 174°14–5′E (29)	194	43	151	0	0	194	0	0	1	23.5	23.3	19:31–20:23	27-Feb	2568	75,545
KH-12-1	27°41–2′N, 169°23–4E (30)	42	9	33	0	0	42	0	0	2	22.1	20.5	18:41–19:32	29-Feb	2486	16,895
KH-12-1	30°15′N, 159°04–5′E (31)	0	0	0	0	0	0	0	0	0	18.9	18.2	19:34–20:25	2-Mar	2420	0
KH-12-1	31°26–7′N, 154°06–7′E (32)	0	0	0	0	0	0	0	0	0	17.7	17.5	18:42–19:33	3-Mar	2323	0
KH-12-1	In total	501	197	304	0	0	501	0	0	0					14,498	34,556

**Table 2:** Comparison of SHCT and HCT between the summer and winter for adults of *Halobates sericeus* (*H.s.*). SHCT: temperature at which semi-heat coma occurred; HCT: temperature at which heat-coma occurred [Mean ± SD(n)]. Specimens were collected 1–14 September 2010 during the science cruise, KH-10-04-Leg 1 (summer) and 26 February–1 March 2012, during the science cruise, KH-12-01-Leg 2 (winter).

SHCT	Summer	Winter
*Females*	36.1 ± 1.9 (62)	33.7 ± 2.3 (18)
*Males*	35.8 ± 2.2 (66)	33.7 ± 1.6 (17)
Total	36.0 ± 2.1(128)	33.7 ± 2.0 (35)
**HCT**	**Summer**	**Winter**
*Females*	36.8 ± 1.2 (62)	35.5 ± 0.9 (18)
*Males*	36.4 ± 1.4 (66)	34.6 ± 0.8 (17)
Total	36.6 ± 1.3 (128)	35.1 ± 0.9 (35)

## References

[1] Andreadis S.S., Athanassiou C.G. (2007). A review of insect cold hardiness and its potential in stored products insect control. Crop Prot..

[2] Harada T. (2003). Hardiness to low temperature and drought in a water strider, *Aquarius paludum* in comparison with other insect groups. Trends Entomol..

[3] Cheng L. (1985). Biology of *Halobates* (Heteroptera: Gerridae). Annu. Rev. Entomol..

[4] Andersen N.M., Cheng L. (2004). The marine insect *Halobates* (Heteroptera: Gerridae): Biology, adaptations distribution, and phylogeny. Oceanogr. Mar. Biol..

[5] Harada T. (2005). Geographical distribution of three oceanic *Halobates* spp.. and an account of the behaviour of H. sericeus (Heteroptera: Gerridae). Eur. J. Entomol..

[6] Harada T., Takenaka S., Sekimoto T., Nakajyo M., Inoue T., Ishibashi T., Katagiri C. (2011). Heat coma as an indicator of resistance to environmental stress and its relationship to ocean dynamics in the sea skaters, *Halobates* (Heteroptera: Gerridae). Insect Sci..

[7] Andersen N.M. (1982). The Semiaquatic Bugs (Hemiptera: Gerromorpha) Phylogeny, Adaptations, Biogeography and Classification.

[8] Pörtner H.O. (2002). Physiological basis of temperature-dependent biogeography: Trade-offs in muscle design and performance in polar ectotherms. J. Exp. Biol..

[9] Peck L.S., Morley S.A., Clark M.S. (2010). Poor acclimation capacities in Antarctic marine ectotherms. Mar. Biol..

[10] Lagerspetz K.Y.H. (2006). What is thermal acclimation?. J. Therm. Biol..

[11] Leroi A.M., Bennett A.F., Lenski R.E. (1994). Temperature acclimation and competitive fitness: An experimental test of the beneficial acclimation assumption. Proc. Natl. Acad. Sci. USA.

[12] Morley S.A., Hirse T., Thorne M.A.S., Pörtner H.O., Peck L.S. (2012). Physiological plasticity, long term resistance or acclimation to temperature, in the Antarctic bivalve, *Laternulae lliptica*.. Comp. Biochem. Physiol. Part A.

[13] Brett J.R. (1956). Some principles in the thermal requirements of fishes. Q. J. Biol..

[14] Schmidt-Nielsen K. (1990). Animal Physiology: Adaptation and Environment.

[15] Jumbam K.R., Jackson S., Terblanche J.S., McGeoch M.A., Chown L. (2008). Acclimation effects on critical and lethal thermal limits of workers of the Argentine ant, *Linepithema humile*.. J. Insect Physiol..

[16] Wilson P.W., Heneghan A.F., Haymet A.D.J. (2003). Ice nucleation in nature: Super cooling point (SCP) measurements and the role of heterogeneous nucleation. Cryobiology.

[17] Harada T., Takenaka S., Sekimoto T., Ohsumi Y., Nakajyo M., Katagiri C. (2011). Heatcoma and its relationship to ocean dynamics in the oceanic sea skaters of *Halobates* (Heteroptera: Gerridae) inhabiting Indian and Pacific Oceans. J. Therm. Biol..

[18] Harada T., Takenaka S., Sekimoto T., Osumi Y., Iyota K., Furutani T., Shiraki T., Nakajo M., Katagiri C., Moku M. (2012). Correlation analysis of heat hardiness and super-cooling point in the oceanic sea skaters, *Halobates*.. Trends Entomol..

[19] Harada T., Takenaka S., Iyota K., Shiraki T., Moku M., Katagiri C., Koštál V. (2013). Supercooling points and heat coma temperatures in four species of oceanic sea skaters of the genus *Halobates* (Heteroptera: Gerridae: Halobatinae). J. Asia-Pac. Entomol..

[20] Cheng L., Shulenberger E. (1980). Distribution and abundance of *Halobates* species (Insecta: Heteroptera) in the eastern tropical Pacific. Fish. Bull..

[21] Furuki T., Umamoto N., Nakajo M., Sekimoto T., Moku M., Katagiri C., Harada T. (2015). Comparative study of cool coma temperature between two populations of oceanic sea skaters, *Halobates sericeus* (Heteroptera: Gerridae), located at 24–25°N and 138°E or 160°E in the Pacific Ocean. Trends Entomol..

[22] Harada T., Sekimoto T., Iyota K., Shiraki T., Takenaka S., Nakajyo M., Osumi Y., Katagiri C. (2010). Comparison of the population density of oceanic sea skater of *Halobates* (Heteroptera: Gerridae) among several areas in the tropical Pacific Ocean and the tropical Indian ocean. Formos. Entomol..

[23] Nakajo M., Sekimoto T., Emi K., Ide R., Wada K., Inoue T., Moku M., Koštál V., Katagiri C., Harada T. (2013). Comparison of temperature preference for habitat among three species of oceanic sea skaters, *Halobates micans*, *Hgermanus* and *H. sericeus*.. Nat. Sci..

[24] Harada T., Osumi Y., Sekimtoto T., Iyota K., Shiraki T., Takenaka S., Takeuchi H., Nakajo M., Tamura T. (2017). The distribution of the oceanic sea skaters, *Halobates* inside and outside the *Kuroshio*.. Open J. Mar. Sci..

[25] Holmstrup M., Zachariassen K.E. (1996). Physiology of cold hardiness in earthworms. Comp. Biochem. Physiol. Part A.

[26] Richard J., Morley S.A., Deloffre J., Pecl L.S. (2012). Thermal acclimation capacity for four Arctic marine benthic species. J. Exp. Mar. Biol. Ecol..

[27] Ma G., Ma C.-S. (2012). Climate warming may increase aphids’ dropping probabilities in response to high temperatures. J. Insect Physiol..

[28] Cooper B.S., Williams B.H., Angilletta M.J. (2008). Unifying indices of heat tolerance in ectotherms. J. Therm. Biol..

[29] Hazell S.P., Neve B.P., Groutides C., Douglas A.E., Blackburn T.M., Bale J.S. (2010). Hyperthermic aphids: Insights into behaviour and mortality. J. Insect Physiol..

[30] Mitchell K.A., Hoffmann A.A. (2010). Thermal ramping rate influences evolutionary potential and species differences for upper thermal limits in *Drosophila*.. Funct. Ecol..

[31] Terblanche J.S., Deere J.A., Clusella-Trullas S., Janion C., Chown S.L. (2007). Critical thermal limits depend on methodological context. Proc. R. Soc. Lond. B.

